# Bacterial adhesion and surface roughness of particulate-filled and short fiber-reinforced composites

**DOI:** 10.1007/s10266-024-00997-z

**Published:** 2024-09-24

**Authors:** L. Lassila, V. Loimaranta, P. K. Vallittu, S. Garoushi

**Affiliations:** 1 Department of Biomaterials Science and Turku Clinical Biomaterial Center, TCBC, Institute of Dentistry, University of Turku, Turku, Finland; 2 Wellbeing Services County of South-West Finland, Turku, Finland

**Keywords:** Short fiber-reinforced composites, Bacterial adhesion, Surface roughness, *Streptococcus mutans*

## Abstract

The objective of the study was to assess the initial adhesion of *Streptococcus mutans* (*S. mutans*) and surface roughness of different particulate-filled (PFC) and short fiber-reinforced (SFRC) composites. Five PFC composites (CeramX Universal, Filtek Universal, Omnichroma, Tetric Prime and Venus Diamond) and four SFRC composites (everX Posterior, everX Flow Bulk, everX Flow Dentin and experimental packable SFRC) were tested in this study. A non-contact 3D profilometer was employed to assess the surface roughness (Ra) of the polished specimens (using 4000-grit abrasive paper). For the bacterial adhesion test, the specimens (*n* = 5/group) were immersed in a solution of *S. mutans* to facilitate initial adhesion. To determine the number of cells on the surfaces of the discs as colony-forming units (CFU), the vials holding the microbial samples were highly agitated using a vortex machine. Subsequently, the samples were diluted multiple times and anaerobically incubated for 48 h at 37 °C on Mitis Salivarius Agar plates (Difco) supplemented with bacitracin. Bacterial adherence assessment was performed using SEM. The data were analyzed using ANOVA. All tested PFC and SFRC composites showed similar adhesion of *S. mutan*. The lowest Ra values (0.26 µm) (*p* < 0.05) were found in the flowable SFRCs (everX Flow Bulk & Dentin), while the highest values (*p* < 0.05) were observed in CeramX and everX Posterior (0.42 µm). Experimental SFRC had comparable Ra value (0.38 µm) than other commercial composites. The presence of short microfibers in the composite appeared to have no adverse effects on the initial adhesion of bacteria or the surface roughness.

## Introduction

Resin composites are the preferred materials for direct restoration due to their ease of application and esthetic benefits [1]. With the growing popularity of resin composites, research in this field has been steadily increasing in recent years [2]. Resin composites have undergone numerous modifications, including alterations to filler size, resin matrix composition, and curing techniques [2]. These improvements have resulted to the production of materials with improved handling/application characteristics, making them appropriate for restoring different kinds of cavities.

Unlike traditional resin composites, which are usually placed in 2 mm layers, bulk-fill resin composites are developed to enable for placement in thicker layers, up to 4–6 mm at a time [3, 4]. Bulk-fill resin composites are reported to have reduced shrinkage stress and increased photo-polymerization levels at more depths, mainly due to enhanced translucency and the inclusion of polymerization modulators [3, 5, 6]. Obtaining the perfect esthetic outcome with direct resin composites necessitates precise layering of various opacities and values, which stands in contrast to the idea of bulk filling, thus compromising several of its benefits. Modern resin composites no longer rely on pigments to achieve color diversity. Instead, they use filler systems that have refractive indices similar to the cured resin matrix [7]. Consequently, they achieve sufficient light diffusivity, yielding what is known as the chameleon effect. This advancement has promoted the creation of one-shade restoratives capable of accommodating a broad spectrum of traditional shades [8].

Another innovative resin composite, incorporating discontinuous glass fiber fillers, offers the advantage of bulk application to reinforce the tooth/composite complex during restoration. Short fiber-reinforced resin composites (SFRC) serve as a viable alternative to dentin in restoring large cavities. By incorporating fiber fillers, these resin composites effectively hinder crack propagation, thereby reducing the likelihood of restoration fractures, a common issue contributing to composite restorations failure [9–12].

The presence of biofilms of bacteria on the teeth’s enamel in the mouth is recognized as the main component that contributes to the development of dental caries [13]. The adherence of microorganisms to restorative materials is crucial in determining the durability of composite restorations in the mouth. Furthermore, the uneven texture of the dental restoration material promotes the accumulation of plaque, perhaps leading to a higher possibility of developing secondary caries [14].

*Streptococcus mutans (S. mutans*), an important contributor to the development of tooth caries, has also been identified as playing a role in the initial generation of dental plaque. The results of in situ as well as laboratory investigations have revealed the existence of *S. mutans* among the bacteria extracted from plaque samples found on both natural and artificial surfaces during the initial stages of caries progression [15, 16]. Nevertheless, it is widely recognized that the initial phase of colonization by an organism involves the attachment of the organism to a surface of the host. Considering this perspective, the assessment of *S. mutans* adhesion and colonization on restorative materials is crucial for determining their clinical performance [15].

According to the literature, SFRC composites have demonstrated good performance in mechanical, loading, optical, wear, and bonding properties compared to many commercial conventional/bulk-fill resin composites [9–12, 17, 18]. However, a question arises regarding the bacterial adhesion of this kind of restorative materials. Will the inclusion of isotropic short-fiber fillers impact the bacterial adhesion and surface roughness of SFRC composites? As far as we know, there is currently no information available about the bacterial adhesion of these materials. Hence, the present investigation aimed to explore the initial adhesion of *S. mutans* and surface roughness among various commercial and experimental SFRC composites, as well as different commercial particulate-filled resin (PFC) composites, including conventional, and one-shade options. The research hypothesis suggested that the type of material used would not impact the surface characteristics of resin composites.

## Materials and methods

Table 1 presents a compilation of the PFC and SFRC resin composites used in the investigation, along with their respective compositions.

**Table 1:** Commercial composite resins used

Material (shade and type)	Manufacturer	Composition
CeramX Universal (A2, conventional)	Dentsply-Sirona, Konstanz, Germany	Methacrylate-modified polysiloxane, dimethacrylate resin, barium–aluminum borosilicate fillers 76 wt.%
Filtek Universal Restorative (A2, conventional)	3 M, St. Paul, MN, USA	AFM, AUDMA, DUDMA, and DDDMA, silane-treated ceramic, silane-treated silica, silane-treated zirconia, 76.5 wt.%
Omnichroma (one-shade, conventional)	Tokuyama Dental Corporation, Tokyo, Japan	UDMA/TEGDMA monomers, spherical SiO2–ZrO2 79 wt.%
Tetric Prime (A2, conventional)	Ivoclar Vivadent, Schaan, Liechtenstein	Bis-GMA, UDMA, Bis-EMA, D3MA barium glass, ytterbium trifluoride, mixed oxide (SiO2/ZrO2) 77 wt.%
Venus Diamond (one-shade, conventional)	Kulzer GmbH (Hanau, Germany	UDMA, TCD-urethaneacrylate. barium, aluminum, silicate glass, 82 wt.%
everX Posterior (transparent, SFRC)	GC Corp, Tokyo, Japan	Bis-GMA, PMMA, TEGDMA, millimeter-scale glass fiber filler, barium glass 76 wt.%
everX Flow Bulk (transparent, SFRC)	GC Corp, Tokyo, Japan	Bis-EMA, TEGDMA, UDMA, short glass microfiber, barium glass 70 wt.%
everX Flow Dentin (SFRC)	GC Corp, Tokyo, Japan	Bis-EMA, TEGDMA, UDMA, short glass microfiber, Barium glass 70 wt.%
Experimental SFRC (one-shade)	Stick Tech-GC member, Finland	Bis-EMA, TEGDMA, UDMA, short glass microfiber, barium glass 78 wt.%

Bis-GMA, Bisphenol-A-glycidyl dimethacrylate; TEGDMA, triethylene glycol dimethacrylate; UDMA, urethane dimethacrylate; TCD, tricyclodecane-urethaneacrylate; Bis-MEPP, bis (p-methacryloxy (ethoxy)1–2 phenyl)-propane; Bis-EMA, ethoxylated bisphenol-A-dimethacrylate; AFM, addition-fragmentation monomer; AUDMA, aromatic urethane dimethacrylate; DDDMA, 1,12-dodecane dimethacrylate; DUDMA, diurethane dimethacrylate; PMMA, poly(methyl methacrylate); wt%, weight percentage

Forty-five disk-shaped specimens, measuring 2 mm in thickness and 6.5 mm in diameter, were fabricated (*n* = 5/group) with a translucent silicone mold. The determination of the sample size was derived from prior studies documented in the literature [19, 20].

A Mylar strip and a 1 mm thick glass slide were placed on top of the silicone mold as part of the specimen preparation process. A gentle force was applied on the glass slide. The application of pressure had two objectives: to eliminate any surplus resin composite from the mold and to guarantee uniformity in the specimen’s thickness. One researcher was in charge of processing all of the specimens.

On both the top and bottom sides, an LED light curing unit (Elipar Deepcure-S, 3 M ESPE, Germany) was used to photo-polymerize the resin composites at an intensity of 1200 mW/cm^2^ for 20 s according to the manufacturer’s recommendations. A calibrator (MARC resin calibrator, BlueLight Analytics, Canada) was used to make sure that the light cure unit was working at the right level before each group was made.

Once the photo-polymerization was done, the specimens were cleaned by running water and rubbing them with 1200-grit silicon carbide abrasive paper (CarbiMet, Buehler, Lake Bluff, IL). The specimen was polished on both surfaces using 2000-grit and 4000-grit FEPA abrasive paper (2000-grit and 4000-grit FEPA) over 20 s with both. An automatic grinding machine (Struers Rotopol-11, Copenhagen, Denmark) was used to do this at 300 rpm under water cooling. Subsequently, the specimens were immersed in water and kept at a temperature of 37 °C for a duration of 48 h prior to conducting the tests.

### Measurement of surface roughness

**Fig. 1:**
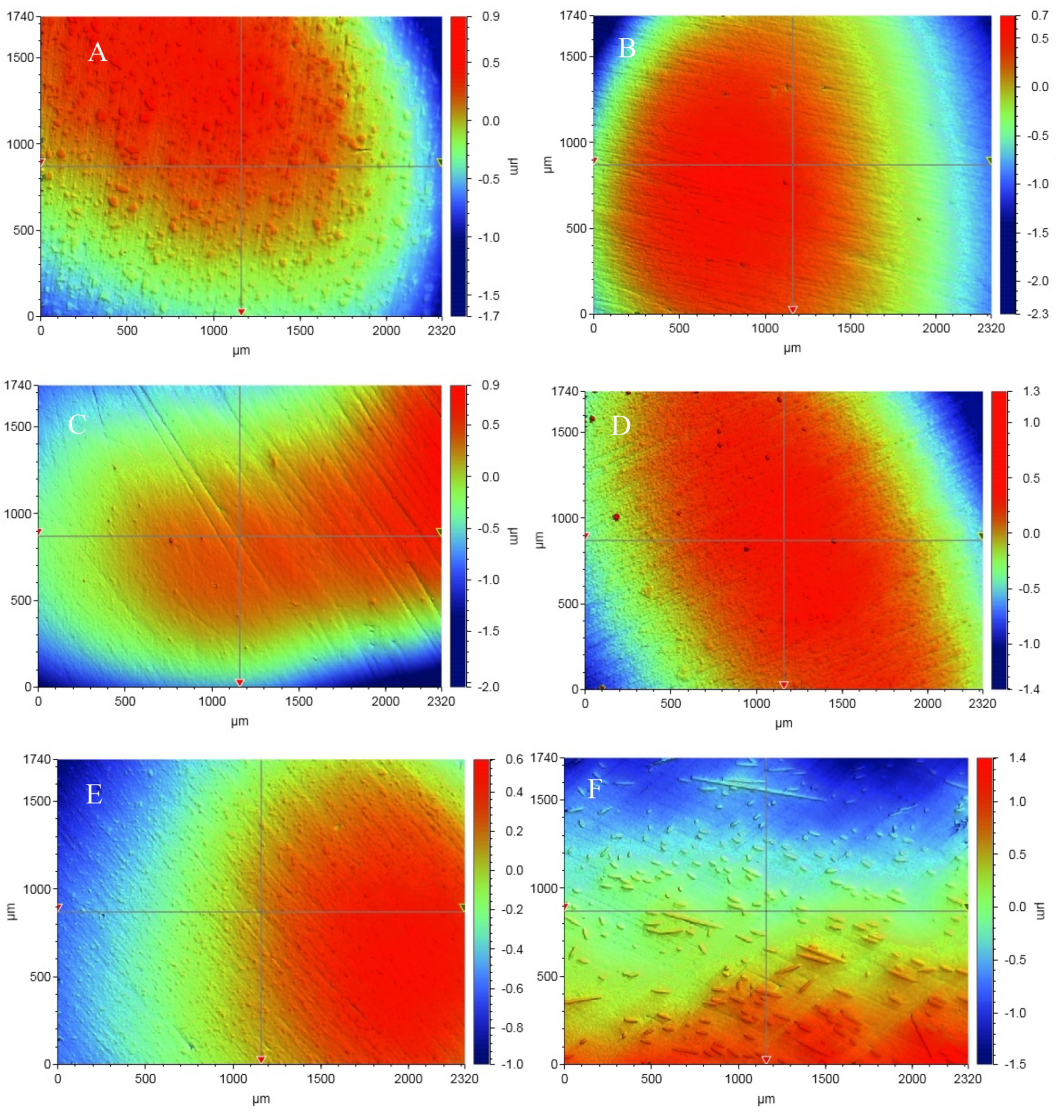

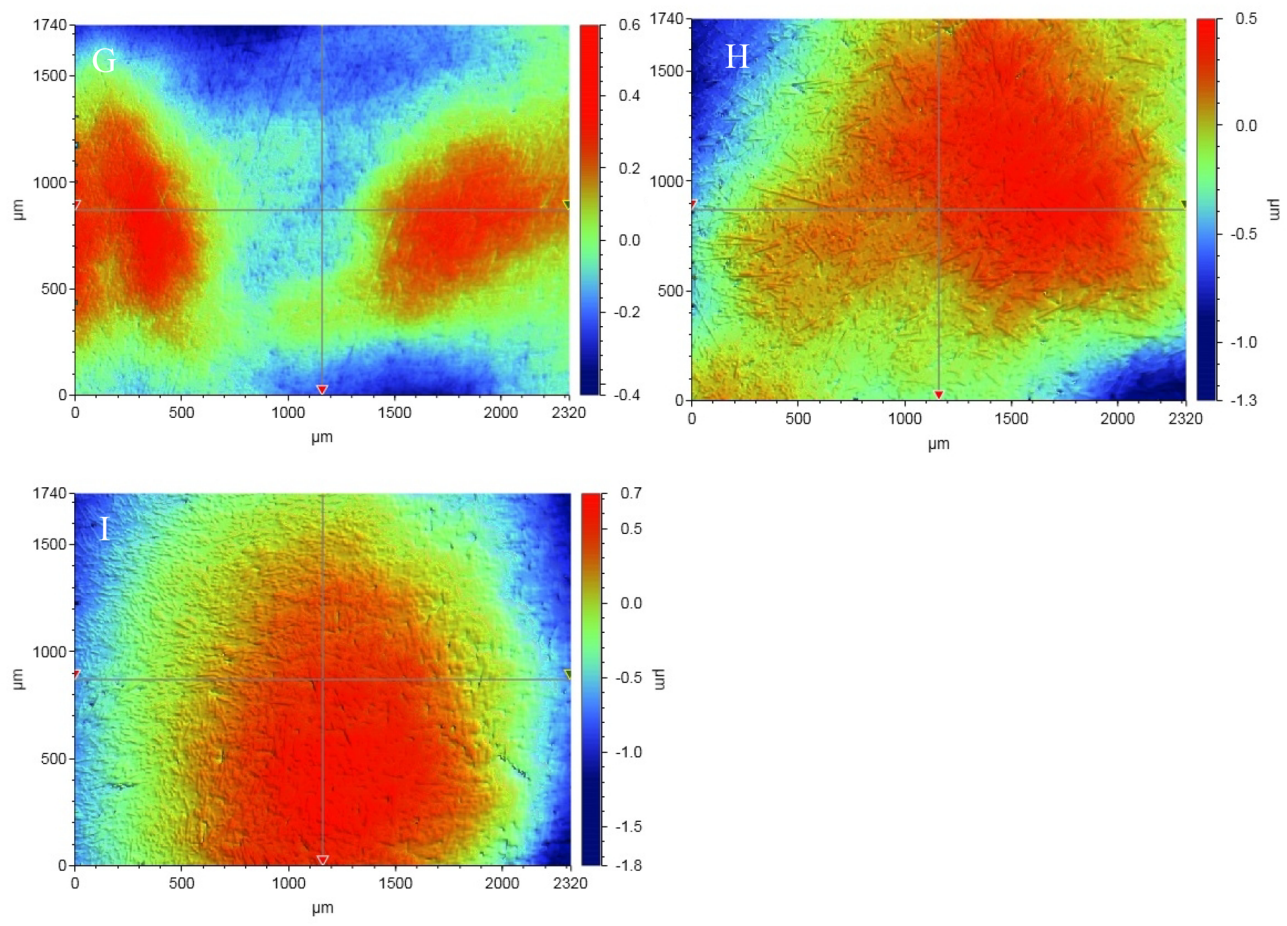
Typical 3D surface profile of investigated resin composites. (**A**) CeramX; (**B**) Filtek; (**C**) Omnichroma; (**D**) Tetric; (**E**) Venus; (**F**) everX P; (**G**) everX FB; (**H**) everX FD; (**I**) SFRC

A 3D non-contact optical profilometer (Bruker Nano GmbH, Germany) was used to assess the surface roughness of every resin composite before the bacterial adhesion test. To generate a 3D depiction of the specimen surfaces, a 5 × objective lens and a 0.5 multiplier were employed. In the VSI/VXI mode, the back scan width was set to 20 µm and the length was set to 60 µm. The experiment was conducted with five specimens for each material. The Vision 64 program was utilized to compute the roughness and surface area metrics. The characteristic of interest, Ra (roughness average), was calculated in the average line inside the sampling length (Fig. 1).

### Cultivation of the microorganism and preparation of cell suspensions

**Fig. 2:**
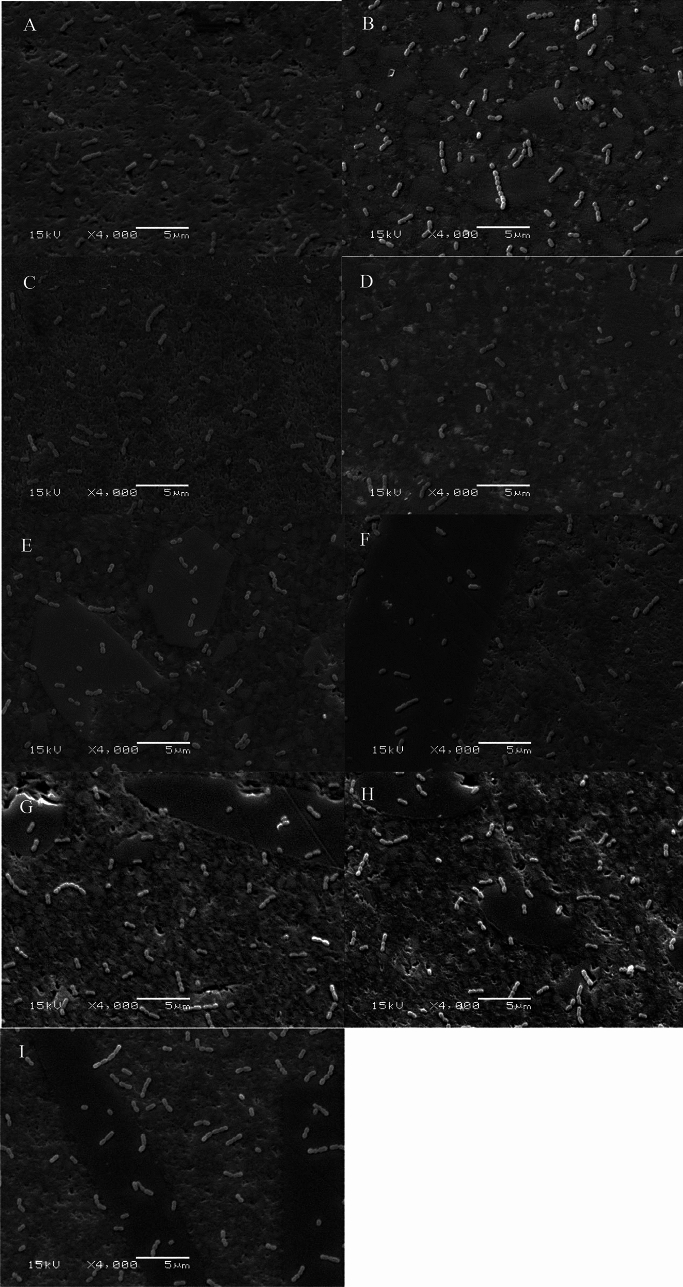
SEM photomicrographs (magnification: 4000 x) of investigated resin composites with adhered *S. mutans* cells. (**A**) Ceram.X; (**B**) Filtek U; (**C**) Omnichroma; (**D**) Tetric Prime; (**E**) Venus; (**F**) everX Posterior; (**G**) everX Flow B; (**H**) everX Flow D; (**I**) Exp. SFRC

*Streptococcus mutans* was cultivated on blood plates (Orion Diagnostica, Espoo, Finland) under anaerobic conditions at 37 °C for 16 h to conduct the adhesion experiments. In such conditions, the cells exist in a state of suspension as single cells, pairs, or triplets, thereby obviating the necessity for further homogenization. The cells were taken off the plates using plastic loops and rinsed two times at a centrifugal force of 10,000* g* for 10 min with phosphate-buffered saline (PBS; containing 137 mM NaCl, 10 mM phosphate, 2.7 mM KCl, pH 7.4). The cells were then mixed with the adsorption buffer at an optical density of 0.35 (A550), which means there were 5 × 10^8^ colony-forming units per milliliter [21, 22]. The cell density was evaluated during the initial adhesion experiments, which showed a uniform distribution of cells on the surfaces of the materials, allowing sufficient room for surface colonization, as evidenced in the scanning electron microscope (SEM) images (Fig. 2).

### Adhesion tests

The adhesion experiments were conducted in accordance with the previously outlined methodology [20, 22]. Five discs from each group (*n* = 45) were initially placed in 2 ml of adsorption buffer that had been mixed with diluted saliva. This was done at the temperature of the room for a duration of 30 min using gentle rolling in 14-ml plastic test tubes with caps that have an inside diameter of 16 mm. The mixing device was tilted 15 degrees to provide continuous liquid coverage of the discs. Once the discs had been pre-incubated, they were washed in 50 ml of saline solution from Orion Diagnostica (Espoo, Finland), which contained 0.9% NaCl. After that, they were put into test tubes that already had 2 ml of the cell suspension that had been made as before. After being submerged in the cell solutions for 30 min, the discs were carefully washed three times with 50 cc of saline solution to remove unbonded bacteria. Subsequently, the cells adhering to one side of the disc were gently removed and transferred to a 0.5 ml solution of transport medium (Tryptic Soy Broth, Difco Laboratories, MI, USA). Before the process began, three applicators were put in a new transport medium called Quick-Stick, which was made by Dentsolv AB in Sweden. These applicators were then used to collect cells by scraping them out of the disc. The applicators’ bristle tips were inserted into the transfer or transport media. During initial trials, subjecting the vials to a single vortex treatment effectively eliminated the bacteria from brushes 1 and 2. However, the number of cells obtained did not go up when the number of scrapings was raised from three to four. Early plaque formed on other materials was collected in the same way [22, 23]. The studies were conducted with three to four replicates and repeated at a minimum of one additional time.

To enumerate cells on the surfaces of the discs as colony-forming units (CFU), the vials containing the microbe samples obtained from the surfaces were vigorously mixed using a vortex machine. The samples were then diluted many times and cultured under anaerobic conditions for 2 days at a temperature of 37 °C on Mitis Salivarius Agar (Difco).

### SEM analysis

The specimens (*n* = 2/per group) used for the adhesion test were immersed in a solution consisting of 2% glutaraldehyde and 2% formaldehyde in phosphate-buffered saline (Orion Diagnostica, Espoo, Finland, pH 7.4) for a duration of 5 min to ensure fixation. Afterward, the objects were rinsed with distilled water and dried using a sequence of increasing concentrations of ethanol: 50% ethanol over 5 min, 70% ethanol over 10 min, two rounds of 96% ethanol over 10 min each, and finally pure ethanol over 5 min. The specimens underwent gold coating using a sputter-coating technique (BAL-TEC SCD 050 Sputter Coater, Balzers, Liechtenstein) and were subsequently examined with an SEM model JSM 5500 (JEOL Ltd. Tokyo, Japan). SEM investigations were performed with an applied voltage of 15 kV.

### Statistical analysis

The data were statistically evaluated using analysis of variance (ANOVA) at a significance level of *p* < 0.05 with SPSS version 13 (Statistical Package for Social Science, SPSS Inc., Chicago, IL, USA). Tukey’s post hoc technique was used to conduct further multiple comparisons.

## Result

**Fig. 3:**
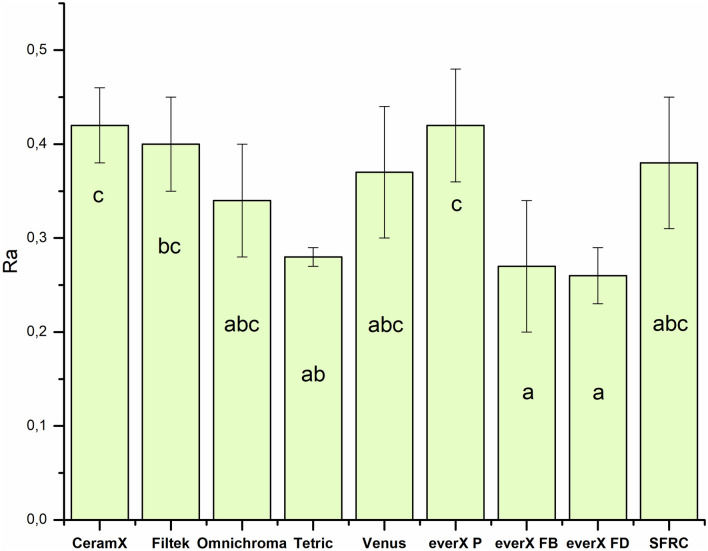
Bar graph illustrating the means of surface roughness Ra (µm) and standard deviations (SD) of the investigated resin composites. Different letters inside bars indicate significant differences (p < 0.05)

The results of surface roughness are presented in Fig. 3. Significant differences in Ra was found based on the type of material (*p* < 0.001). The lowest Ra values (0.26 µm) were found in the flowable SFRCs (everX Flow Bulk & Dentin), while the highest values were observed in CeramX and everX Posterior (0.42 µm). Experimental SFRC composite had comparable Ra value (0.38 µm) than other commercial resin composites.

The 3D images of polished specimens are depicted in Fig. 1. Short fibers were clearly visible in the everX Posterior resin composite, not in other SFRC composites. However, the absence of fiber protrusion was noted, when they were polished with the resin matrix.

SEM analysis (Fig. 2) revealed a range of filler/fiber diameters present within each tested resin composite, thereby providing a justification for the varied surface roughness measurements.

**Fig. 4:**
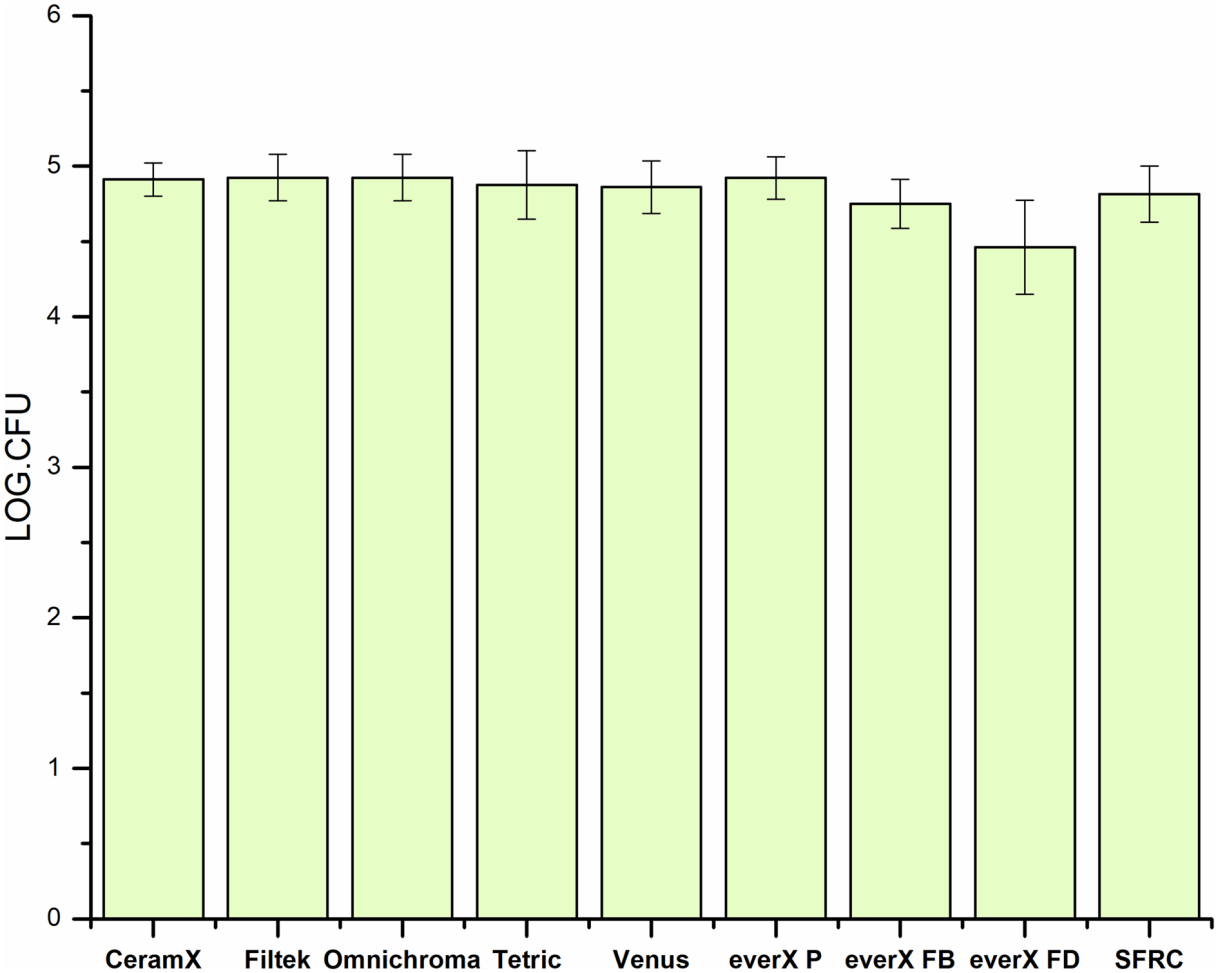
The number of adhered *S. mutans* cells on the investigated resin composites with standard deviations (SD)

**Fig. 5:**
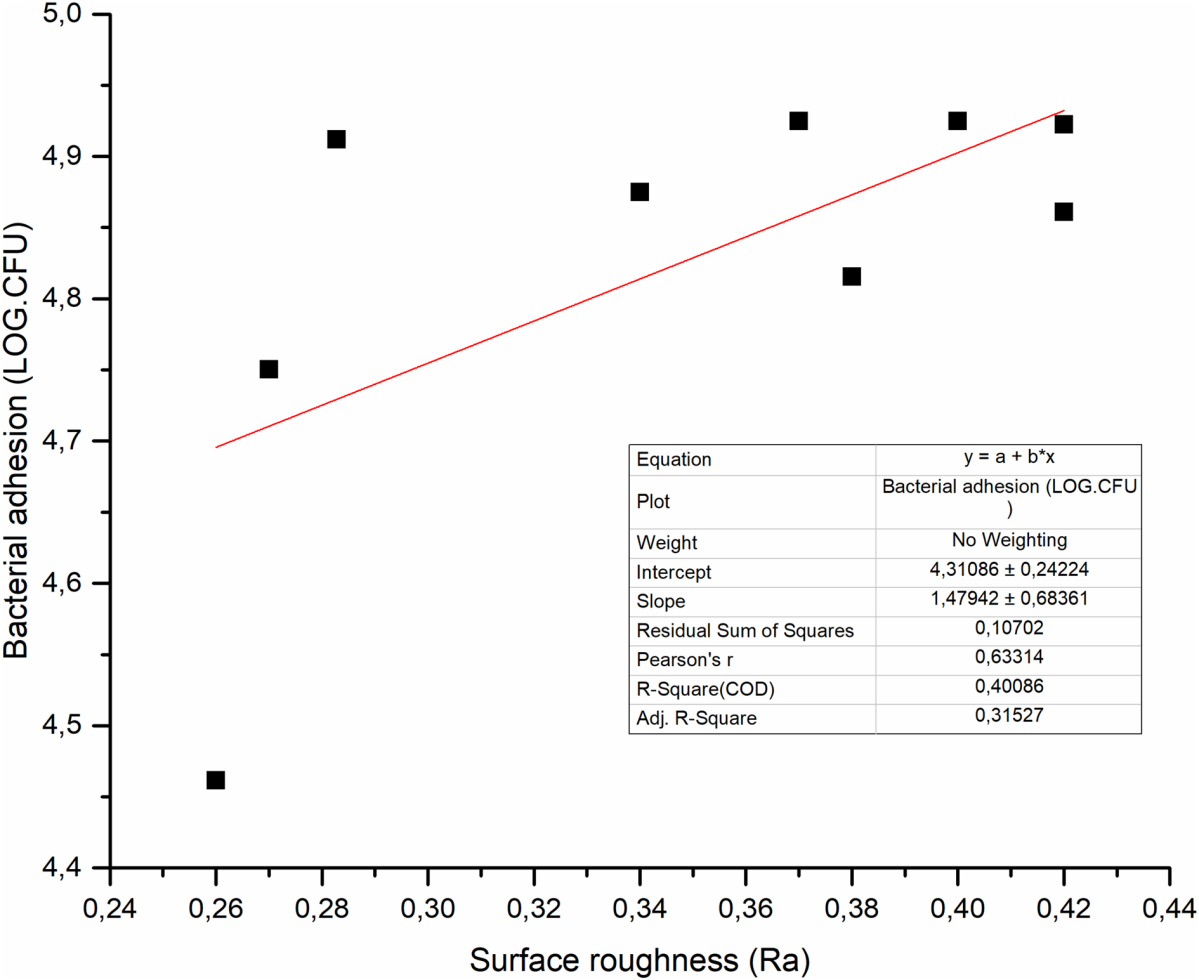
Linear regression between measured bacterial adhesion of the materials and surface roughness of the tested specimens

SFRC and other tested PFC resin composites demonstrated equal adhesion of *S. mutans* (Fig. 4). There was a weak correlation (*R*^2^ = 0.4) between *S. mutans* adhesion and surface roughness (Fig. 5).

## Discussion

The first step in the development of secondary caries typically involves the formation of bacterial colonization by *S. mutans*. The present research looked at how *S. mutans* adhered to various SFRC and PFC resin composites relying on their surface roughness.

Our research hypothesis proposed that the type of material would not affect the tested surface characteristics of resin composites. However, our findings indicate that *S. mutans* did not demonstrate any significant variation in adhesion between experimental and commercial SFRC composites compared to other investigated PFC resin composites (Fig. 4), despite differences in surface roughness (Fig. 3). Therefore, we partially accept our null hypothesis.

Our SEM analysis revealed that bacteria were uniformly distributed throughout the composite matrix. This matrix is filled with filler particles that possess similar hydroxyl groups (–OH) and surface energy as glass fiber fillers. In other words, bacterial adhesion was uniformly distributed between the glass fibers and the polymer matrix, suggesting that both components attract bacterial adhesion equally. It is worth noting that earlier studies have demonstrated that the *S. mutans* adhesion to surfaces increases when the surface’s free energy increases [22–24]. Nevertheless, it was shown that glass fibers with higher surface energy exhibited similar adhesion to *S. mutans* compared to different filling and prosthetic materials that had lower surface energy.

The results of this research align with the investigations conducted by Lassila et al. and Tanner et al., which demonstrated that, when it comes to plaque accumulation and *S. mutans* adherence, glass fiber-reinforced composites (FRC) are similar to PFC resin composites [22, 23]. One study demonstrated a higher level of bacterial adhesion to the surface of glass fibers compared to the polymer matrix [24]. However, in this particular investigation, the matrix consisted solely of an autopolymerized denture base resin containing residual monomers, such as methyl methacrylate and its oxidized counterpart, formaldehyde, which can have cytotoxic effects [25]. Additionally, the existence of –OH on the glass fibers’ surfaces can significantly influence bacterial adhesion compared to the acrylic matrix. Consequently, in their investigation, *S. mutans* exhibited a higher affinity for the glass fiber surface.

The results of our investigations demonstrated that surface roughness was weakly correlated with *S. mutans* adhesion (Fig. 5). Eick et al. showed that there was no relationship between the roughness of a restorative material surface and the quantity of colony-forming units (CFU) of *S. mutans* [26]. Weitman and Eames, as well as Shintani and her colleagues, found that there was no notable variation in the accumulation of plaque on surfaces that were polished using different techniques, as long as the obtained Ra values were within the range of 0.7–1.4 µm [27, 28].

In their assessment of surface roughness and adherence of *S. mutans* on various PFC microhybrid, nanohybrid, nanofill, and bulk-fill resin composites, Cazzaniga et al*.* [20] and Pereira et al*.* [29] used a variety of polishing procedures. Their research indicated that there was no notable difference in surface roughness across nanohybrid, nanofill, and bulk-fill resin composites. Nevertheless, the researchers found that compared to the bulk-fill and conventional resin composites, the nanofill resin composite had far lower bacterial adherence. Therefore, material composition significantly influenced *S. mutans* biofilm formation, but finishing and polishing procedures had no significant impact according to them.

On the contrary, several other published investigations propose that the surface roughness of dental materials plays a crucial role in biofilm formation and bacterial adherence [30–32]. They have significantly elevated surface roughness values in materials with the highest bacterial adhesion (0.6–1.5 μm). When the surface roughness levels go above a certain level, the bacterial adhesion to materials increases. All specimens in our investigation were polished using a laboratory machine, which reportedly produced better results than chair-side polishing in terms of gloss and surface roughness [33, 34].

Figure 3 demonstrates substantial variations in the Ra values across the used resin composites. Compared to the tested one-shade PFC resin composites, the experimental SFRC composite had an identical Ra value. On the other hand, commercial flowable SFRC composites (everX Flow Bulk & Dentin) revealed lower Ra value than other tested resin composites, while the highest Ra values were observed in CeramX and everX Posterior. Several factors contribute to surface roughness. These include matrix characteristics, inorganic particle/fiber proportion and size, particle/fiber exposure, and air bubble formation during material preparation. Based on manufacturer data and SEM images (Fig. 2), everX Posterior, with a fiber diameter of 15 µm, and CeramX, containing pre-polymerized filler (approximately 15 µm) [35], exhibit higher average particle/fiber sizes. In contrast, the largest possible filler/fiber size in everX Flow and Tetric is approximately 5 µm, which could contribute to the reduced roughness observed in these resin composites [9, 36]. Moreover, it seems that the composition of filler particles in resin composites significantly influences their polishability. When particulate fillers are significantly dense or possess greater hardness compared to the resin matrix, the resin matrix might experience specific abrasion during polishing, resulting in the appearance of filler particles on the surface [37]. Bayne and his associates suggest that while the quantity and size of filler are relevant, the arrangement of filler particles and the spacing between them are critical factors in ensuring surface protection for composites [38]. Nevertheless, the Ra values of all examined resin composites (PFC and SFRC) fell within the clinically acceptable range and the threshold of differences was very low (0.26–0.42 µm). Kaplan and his team mentioned that Ra values below 10 µm cannot be detected clinically [39].

The methodological limitation with the way this study was done is that the cells were scraped and there were no labels on them because we wanted to get samples from both sides of the material. The scraping process was thought to be repeatable because there were few standard deviations between the repeats. This also made it possible to look at the same material under a scanning electron microscope and count the number of colony-forming units (CFUs). A negative control group for bacterial adhesion tests was absent, and this aspect will be assessed in the near future. Another limitation was the utilization of only one laboratory system for the polishing technique.

Further research is required to assess the long-term surface characteristics of the experimental packable SFRC resin composite.

## Conclusion

According to the study’s results, the incorporation of short microfibers into the resin composite appeared to have no adverse effects on the initial adhesion of bacteria or the surface roughness.

## Data Availability

The data presented in this study are available on reasonable request from the corresponding author.
